# A Meta-Analysis of Concurrent Chemoradiotherapy for Advanced Esophageal Cancer

**DOI:** 10.1371/journal.pone.0128616

**Published:** 2015-06-05

**Authors:** Li-Li Zhu, Ling Yuan, Hui Wang, Lin Ye, Gui-Ying Yao, Cui Liu, Niu-Niu Sun, Xiao-Jing Li, Shi-Cong Zhai, Ling-Juan Niu, Jun-Bo Zhang, Hong-Long Ji, Xiu-Min Li

**Affiliations:** 1 School of Nursing, Xinxiang Medical University, Henan, China; 2 Department of Radiation, Henan Cancer Hospital, Zhengzhou, Henan, China; 3 The Affiliated Cancer Hospital of Zhengzhou University, Zhengzhou, Henan, China; 4 Ontario Cancer Institute, University of Toronto, Toronto, Ontario, Canada; 5 Department of Oncology, The Third Affiliated Hospital, Xinxiang Medical University, Henan, China; 6 Department of Molecular and Cellular Biology, University of Texas Health Science Center at Tyler, Tyler, Texas, United States of America; 7 Center for Cancer Research, Xinxiang Medical University, Henan, China; NIH, UNITED STATES

## Abstract

**Background:**

Concurrent chemoradiotherapy is a standard treatment for local advanced esophageal cancer, but the outcomes are controversial. Our goals were to compare the therapeutic effects of concurrent chemoradiotherapy and radiotherapy alone in local advanced esophageal cancer using meta-analysis.

**Methods:**

MEDLINE, EMBASE and the Cochrane library were searched for studies comparing chemoradiotherapy with radiotherapy alone for advanced esophageal cancer. Only randomized controlled trials were included, and extracted data were analyzed with Review Manager Version 5.2. The pooled relative risks (RR) and their 95% confidence intervals (CI) were calculated for statistical analysis.

**Results:**

Nine studies were included. Of 1,135 cases, 612 received concurrent chemoradiotherapy and 523 were treated with radiotherapy alone. The overall response rate (complete remission and partial remission) was 93.4% for concurrent chemoradiotherapy and 83.7% for radiotherapy alone (*P *= 0.05). The RR values of 1-year, 3-year, and 5-year survival rates were 1.14 (95% CI: 1.04 - 1.24, *P *= 0.006), 1.66 (95% CI: 1.34 - 2.06, *P* < 0.001), and 2.43 (95% CI: 1.63 - 3.63, *P *< 0.001), respectively. The RR value of the merged occurrence rate of acute toxic effects was 2.34 (95% CI: 1.90 - 2.90, *P* <0.001). There was no difference in the incidence of late toxic effects, which had an RR value of 1.21 (95% CI: 0.96 - 1.54, *P* = 0.11). The RR level of persistence and recurrence was 0.71 (95% CI: 0.62 - 0.81, *P <0*.*001*), and for the distant metastasis rate, the RR value was 0.79 (95% CI: 0.61 - 1.02, *P* = 0.07).

**Conclusions:**

Concurrent chemoradiotherapy significantly improved overall survival rate, reduced the risk of persistence and recurrence, but had little effect on the primary tumor response, and increased the occurrence of acute toxic effects.

## Introduction

Esophageal carcinoma is the eighth most common malignancy worldwide, with an estimated 482,300 new cases each year, and serious impacts on both patient survival and quality of life [1, 2]. Esophagectomy has long been established as the primary treatment modality, and the 5-year survival rate after operation is reported to be 90% with early detection and treatment. However, most patients diagnosed with esophageal cancer are already in the advanced stages of the disease, with only about 20% of cases being resectable [3, 4]. Radiotherapy (RT) plays an important role in advanced esophageal cancer, but the outcome is unsatisfactory due to poor local control and distant metastasis, with a 5-year survival rate of about 10% [5, 6]. Autopsies have confirmed that over 50% of local advanced esophageal cancer patients die of distant or extensive lymph node metastasis. Therefore, RT alone has limited success [7]. In view of this, chemoradiotherapy (CRT) of esophageal cancer has garnered increasing interest, as the combined effects of radiotherapy and chemotherapy may be synergic and complementary for local control and for preventing distant metastasis, thereby enhancing survival.

A number of combinations of RT with chemotherapy have been studied. One is neoadjuvant CRT, in which patients receive chemotherapy and radiation briefly before surgery. The other is concurrent chemoradiotherapy (CCRT), in which patients receive chemotherapy and radiotherapy at the same time, without any surgery. The Radiation Therapy Oncology Group (RTOG) trial, designated RTOG 85–01, showed that CCRT had the best outcomes [8]. Comparing CCRT with RT, the tumor recurrence rate was 45% versus 59% (*P* < 0.001), while the distant metastasis rate was 21% versus 37% (*P* < 0.001). Additionally, the 2-year and 5-year survival rates were 36% versus 26% (*P* < 0.001) and 10% versus 0% (*P* < 0.001) [8]. Two randomized controlled trials (RCTs) confirmed that CCRT rather than sequential chemoradiotherapy resulted in improved patient survival rate in advanced esophageal cancer [9, 10]. Consequently, CCRT is the standard of care for unresectable advanced esophageal carcinoma in the United States and Europe.

However, some clinical trial results suggested that CCRT was unpromising [8–12]. For example, in one RCT, 70 patients were randomly assigned to a CCRT group (34 patients) or RT alone group (36 patients) [13]. The median survival in the CCRT and RT groups was 170 and 144 days, respectively (*P* > 0.05). Herskovic et al. found that in a CCRT group, 44% of patients suffered severe side effects, and 20% suffered life-threatening side effects, while the rates were 25% and 3% for the RT group (*P* < 0.001) [14]. Seung et al. reported increased incidence of respiratory esophagitis of grade II or above, a complication that was difficult for patients to cope with and affected the prognosis [15]. Therefore, whether CCRT actually increases the survival rate in patients with advanced esophageal carcinoma remains controversial.

Although there have been prior meta-analyses on chemoradiotherapy for esophageal carcinoma, those studies focused on adjuvant chemoradiotherapy or neoadjuvant chemoradiotherapy for resectable esophageal cancer [16–19]. In contrast, our analysis assesses the effectiveness of CCRT, and involves patients with advanced disease with unresectable carcinomas.

In order to provide reliable evidence for the effectiveness of CCRT in advanced esophageal cancer, a meta-analysis of clinical studies was performed with a focus on the evaluation of primary tumor response, survival rate, toxicities and patterns of failure compared with patients treated with conventional RT alone.

## Materials and Methods

### Search strategy

Computerized bibliographic searches were performed to identify all eligible published literature between May 1991 and December 2014. MEDLINE, EMBASE and the Cochrane library were the primary sources. The core search consisted of terms related to cancer sites (esophageal OR esophagus OR oesophagus) and descriptions of cancer (cancer OR neoplasm OR carcinoma OR tumor). These were combined with specific terms for treatments (chemoradiotherapy OR chemoradiation OR radiochemotherapy OR chemotherapy OR radiotherapy OR radiation). There were 11523 studies identified by electronic search using keywords. Two additional articles were identified by manual searching of the reference sections of topical papers. Among these studies, 452 of the studies published in English involved randomized controlled trials. From this set, titles and abstracts of 426 articles obtained were screened by C. Liu, G.-Y. Yao, X.-J. Li and N.-N. Sun to exclude those not relevant to the study. The remaining 26 full-text articles were read carefully and nine studies were saved for further analysis (Table 1). To ensure the reliability of the literature search and to avoid bias, trials were chosen by two independent researchers, L. Yuan and L. Ye.

**Table 1:** 

Searching process	Number of studies
Studies identified by electronic search using keywords	14676
Articles identified by manual search that met the criteria	2
Published between May 1991 and December 2014	11523
Randomized controlled trials of article type	452
Titles and abstracts excluded by screening	426
Full-text screened for detailed analysis	26
Studies excluded following detailed analysis	17
1. Patients were in the early stages of cancer	2
2. Patients had undergone esophagectomy	2
3. Studies had fewer than 50 samples	2
4. Outcomes did not include complete data	10
5. Duplicate study	1
Studies included following detailed analysis	9

### Inclusion and exclusion criteria

All eligible studies conformed to the following criteria: (1) They compared effects of CCRT and RT alone on advanced esophageal cancer, and were published in English. (2) RCTs had a total of more than 50 samples, follow-up rates above 90% and follow-up periods not less than 3 years. (3) Esophageal squamous cell carcinoma (SCC) and adenocarcinoma (AC) were confirmed by histological cytology. (4) There was no statistically significant difference in patient or disease features, including sex, age, type of pathology, and tumor stage between the two groups (*P* > 0.05). (5) Studies had obtained informed consent. (6) Outcomes included overall response rate, 1-year, 3-year, and 5-year survival rates, rate of acute and late toxic effects, rate of persistence and recurrence, and rate of distant metastasis.

The following studies were excluded: (1) Patients were in the early stages of cancer, had undergone esophagectomy or had chemotherapy contraindications; (2) studies did not involve RCTs; or (3) any studies that did not include the survival rate, or the rates of recurrence or distant metastasis.

### Quality assessment

According to the Cochrane Handbook for Systematic Reviews of Interventions [20], all RCTs should be assessed on three fronts: blinding, randomization, and allocation concealment. If all of these criteria are met, there is a low risk of bias. If one or more criteria are partly met, there is a moderate risk of bias. If one or more criteria are not met, there is a high risk of bias. If discrepancies arise while assessing RCTs, a consensus should be reached by discussion. All trials identified in our meta-analysis were randomized and controlled. However, most of the trials did not include clear descriptions regarding blinding and allocation concealment. Thus, the studies have a moderate risk of bias. Discrepancies were resolved by L.-L. Zhu, L. Yuan, and L. Ye.

### Data processing and statistical methods

For each study, we put the following extracted data in the Excel database: title of the study, first author and location (country), the date of publication and journal title, clinical data including age of patients, location of tumor, survival rate and so on. Data extraction was performed by S.-C. Zhai, L.-J. Niu, and J.-B. Zhang. The analysis was performed with Review Manager Version 5.2, and Q statistics were applied to test the heterogeneity of the qualifying studies, with *P* < 0.05 indicating heterogeneity. The I^2^ statistic represents the percentage of the total variability across studies that are due to heterogeneity. I^2^ values of 25%, 50%, and 75% corresponded to low, moderate, and high degrees of heterogeneity, respectively [21]. When either moderate or high heterogeneity was observed, the random-effect model was used. Alternatively, the fixed-effect model was used.

The 1-year, 3-year, and 5-year survival rates, the rates of acute and late toxic effects, rates of persistence and recurrence, and the rate of distant metastasis were estimated using the risk ratio (RR) or the risk difference (RD), with their corresponding 95% confidence intervals (CI) and *P* values. RR forest distribution maps were drawn. The impact of publication bias was assessed by observing the symmetry of funnel plots [22], with the Begg adjusted rank correlation test and Egger’s test. *P* ≤ 0.05 was considered statistically significant.

## Results

### Features of RCTs

Nine randomized studies in the United States, Canada, England, China, India, and Iran were carried out from 1982 to 2005 and the reports were published between 1991 and 2013. These nine RCTs included 1,135 patients [8, 14, 23–29] (Table 2). Of these, 612 patients received CCRT and the remaining 523 patients received RT alone. The most common tumor histology was SCC (97.4%), and the remainder was adenocarcinoma (2.6%). The median age (weighted by trial size) for the CCRT group was 61 (range 24–70), and 60 (range 34–76) for the RT group. No standard approach was used to compare the age of participants between studies, but there were no major discrepancies in the age cohort recruited. All patients were at the T1-3N0-1 stage of the disease. The main features of the trials included in the meta-analysis were listed in Table 2.

**Table 2:** 

First author (year)	Size (CCRT/RT alone)^a^	Type	Location (Upper/middle/lower)	Treatment		Country
				CCRT	RT alone	
AraujoCM 1991	28/31	SCC^b^	NR	5-FU + mitomycin + bleomycin CF 50Gy	CF 50Gy	Canada
CooperJS 1999	61/62	SCC/AC (108/15)	NR	DDP+5-FU CF 50Gy	CF 50Gy	USA
GaoXS 2002	40/41	SCC	22/48/11	DDP CF + LCAF 40Gy	CF + LCAF 40Gy	China
HanJH 2012	65/65	SCC	67/59/5	nedaplatin+5-FU CF 64-66Gy	CF 64-66Gy	China
Herskovic A 1992	61/60	SCC/AC (111/10)	23/59/39	DDP+5-FU CF 50Gy	CF 50Gy	England
KumarS 2007	65/60	SCC	23/20/22	DDP CF + LCAF 50-64Gy	CF+LCAF 50-64Gy	India
Mirinezhad SK 2013	175/92	SCC/AC (253/14)	35/94/138	DDP+5-FU DRT 40-44Gy	DRT 40-44Gy	Iran
ShengW 2011	63/65	SCC	66/39/13	Capecitabine CF + LCAF 64-69Gy	CF + LCAF 64-69Gy	China
ZhaoKL 2005	54/57	SCC	37/70/4	DDP+5-FU CF + LCAF 68.4Gy	CF + LCAF 68.4Gy	China

^a^ The patients in the combination group (CCRT) were treated with radiotherapy and chemotherapy at the same time.

^b^ Abbreviations: SCC, squamous cell carcinoma; AC, adenocarcinoma; NR, no report; 5-FU, 5-fluorouracil; DDP, cisplatin; CF, conventional fraction radiotherapy; LCAF, late course accelerated fractionation radiotherapy; Gy, Gray.

### Assessment of the overall response rate

**Fig 1:**
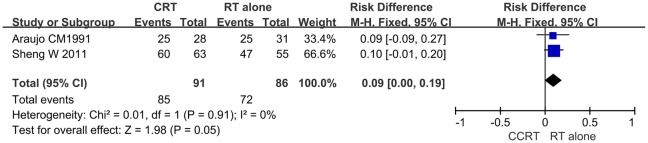
Forest plot comparing primary tumor response rates for the CCRT group and the RT alone group. The blue squares represent study-specific risk difference (RD), the horizontal line represents 95% confidence intervals (CIs), and the black diamond represents summary risk difference.

The primary tumor response was used to evaluate short-term therapeutic effects. Thoracic CT scans, barium meal, and ultrasound imaging were performed at three months following the initiation of treatment. Based on revised RECIST guidelines [30], the treatment responses were divided into complete response (CR), partial response (PR), stable disease (SD), and progressing disease (PD). CR is defined as the disappearance of all target lesions, and any pathological lymph nodes (whether target or non-target) must have a reduction in the short axis to <10 mm. A PR is defined as having at least a 30% decrease in the sum of diameters of target lesions, taking as reference the baseline sum diameters. The objective response (CR + PR) is the primary endpoint. Two RCTs [27, 28] were included in the meta-analysis. The total response rates (CR + PR) of the CCRT and RT alone groups were 93.4% and 83.7%, respectively. The RD was 0.09 (95% CI: 0.00–0.19, *P* = 0.05), indicating that there was a difference in the short-term therapeutic outcomes of CCRT versus RT alone in advanced esophageal cancer (Fig 1).

### Overall survival rate

**Fig 2:**
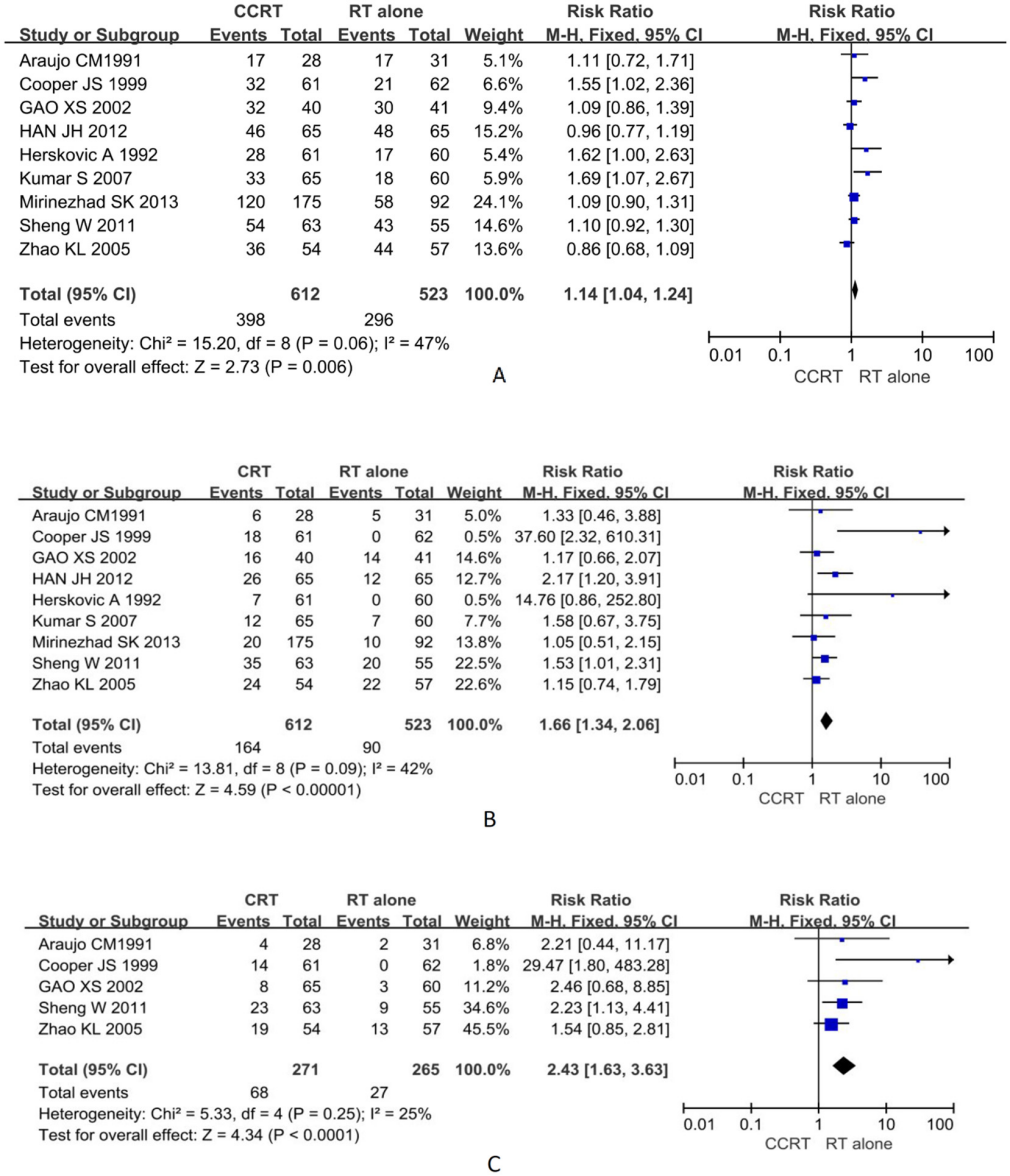
Forest plot comparing survival rates for the CCRT group and the RT alone group. The blue squares represent study-specific relative risks (RR), the horizontal lines represent 95% CIs, and the black diamonds represent the summary of RR. (A) Comparison of 1-year survival rate. (B) Comparison of 3-year survival rate. (C) Comparison of 5-year survival rate.

All nine RCTs reported 1-year and 3-year overall survival rates [8, 14, 23–29], with I^2^ statistics of heterogeneity of *P* = 0.06 and *P* = 0.09, respectively, indicating that there was no heterogeneity across the included RCTs. Thus, the fixed-effect model was selected for the pooled analysis. The RR value, expressed as CCRT versus RT alone, was 1.14 (95% CI: 1.04–1.24, *P* = 0.006) for the 1-year survival rate (Fig 2A). The 3-year survival rate RR value of 1.66 (95% CI: 1.34–2.06, *P* <0.001) was statistically significant (Fig 2B). Five of the nine RCTs reported the 5-year survival rate [8, 23, 26–28], for which the RR value of 2.29 (95% CI: 1.54–3.39, *P <0*.*001*) was statistically significant (Fig 2C). No evidence of publication bias was detected by Egger 's test in the 1-year (t = 2.31, *P* = 0.054), 3-year (t = 2.37, *P* = 0.051), or 5-year survival rates (t = 2.38, *P* = 0.098).

### Toxic effects

**Fig 3:**
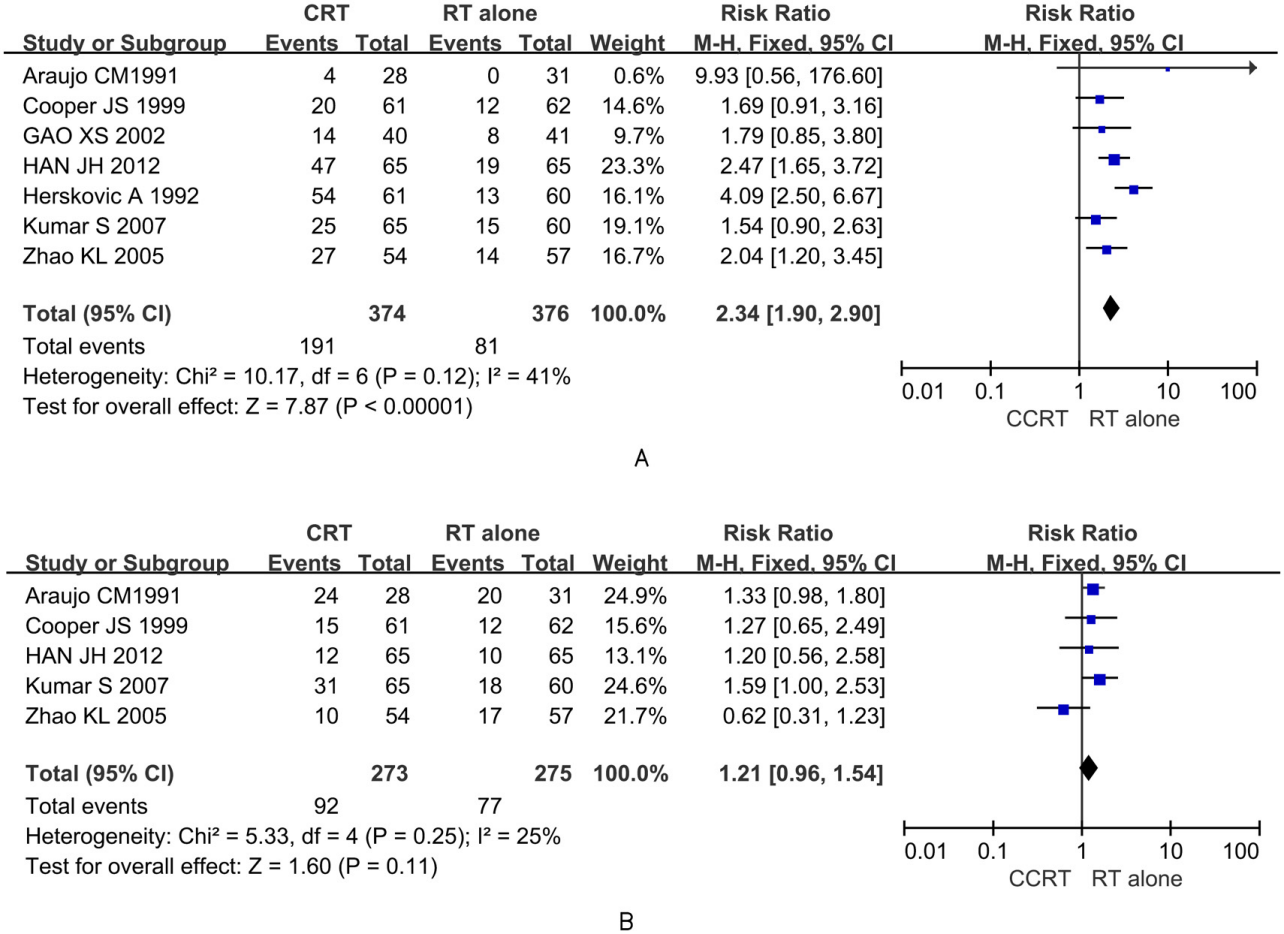
Forest plots comparing rates of toxic effects for the CCRT group and RT alone group. (A) Rate of acute toxic effects. (B) Rate of late toxic effects.

Acute and late toxic effects were evaluated according to the WHO and RTOG criteria [31]. Acute toxic effects (grades 0–4) are defined as occurring in the first three months after radiation. Seven RCTs reported grade 2 or higher serious side effects in patients including nausea or vomiting (8.73%), leucopenia (14.39%) and radiation esophagitis (25.63%). The occurrence of acute toxicities was higher in the CCRT group than in the RT alone group (Fig 3A), with an RR value of 2.34 (95% CI: 1.90–2.90, *P <0*.*001*), and no publication bias was found (*t* = 0.13, *P* = 0.903). Late toxic effects (grades 0–5) were evaluated in patients followed for more than three months after treatment, with radiation—induced esophagitis and pneumonia comprising the majority of occurrences, although esophageal stenosis and pulmonary fibrosis occurred in a few patients. Five of the RCTs referred to late complications of grade 2 or higher [8, 23, 24, 28, 29], with an RR value of 1.21 (95% CI: 0.96–1.54, *P* = 0.11) for CCRT versus RT, with no observed statistical difference (*P* > 0.05) (Fig 3B). Additionally, no publication bias was detected by Egger 's test (*t* = -0.96, *P* = 0.409).

### Reasons for treatment failure

#### Persistence and recurrence

**Fig 4:**
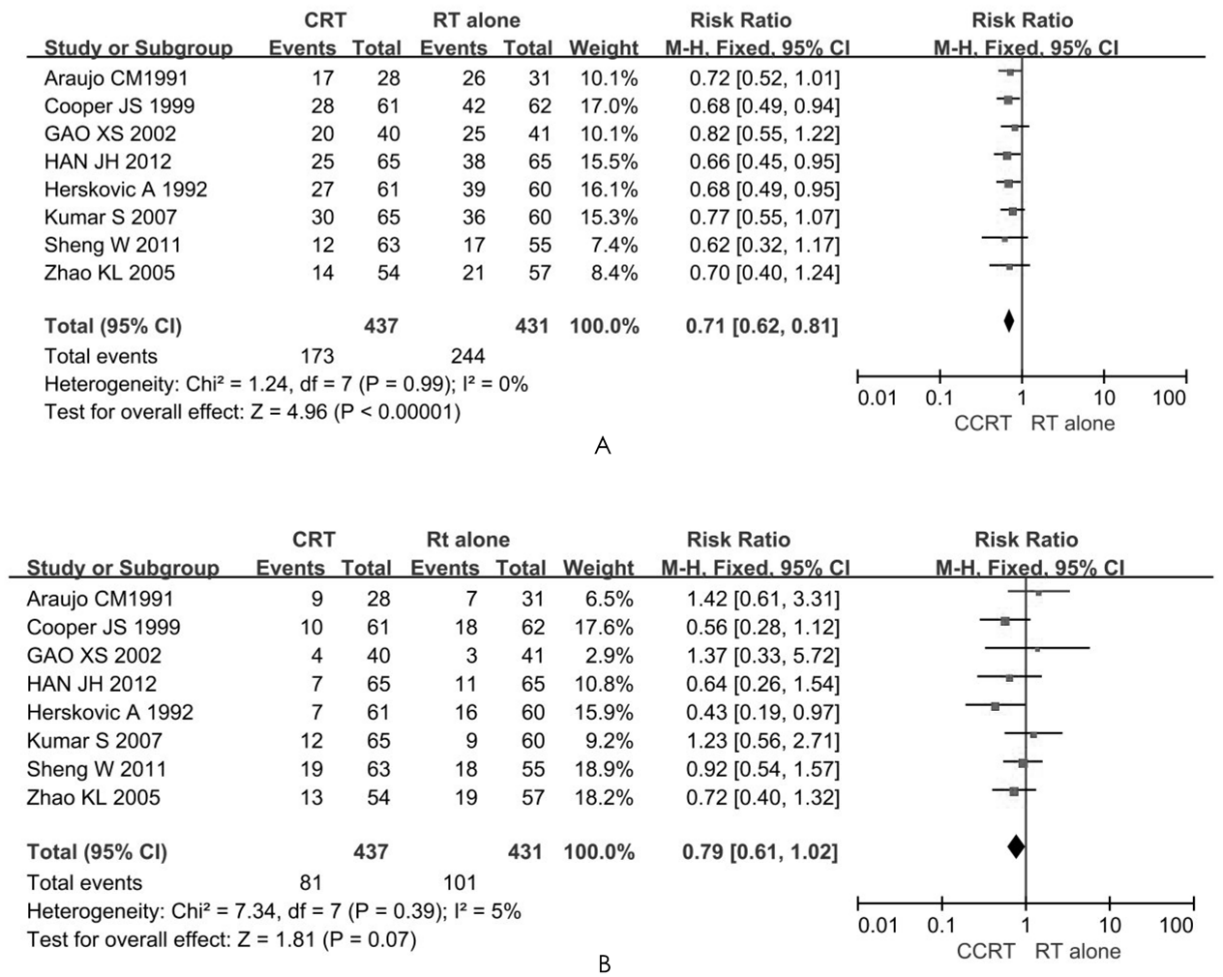
Forest plots comparing rates of (A) persistence and recurrence, and (B) distant metastasis, between the CCRT group and RT alone group.

When a tumor remains or reappears at the site of the primary lesions within six months after radiation, it is said to be persistent, whereas beyond six months it is considered recurrent [32]. Eight RCTs investigated these occurrences of treatment failure [8, 14, 23, 24, 26–29]. Patients treated by concurrent chemoradiotherapy had a lower incidence (17%) of failure, and the overall RR was 0.71 (95% CI: 0.62–0.81, *P <0*.*001*), obtained with absence of heterogeneity (I^2^ = 0%). Therefore, the difference between the CCRT group and the RT alone group was significant (*P* < 0.05) (Fig 4A). Using Egger's test, no publication bias was found (*t* = -0.55, *P* = 0.604).

#### Distant metastasis

**Fig 5:**
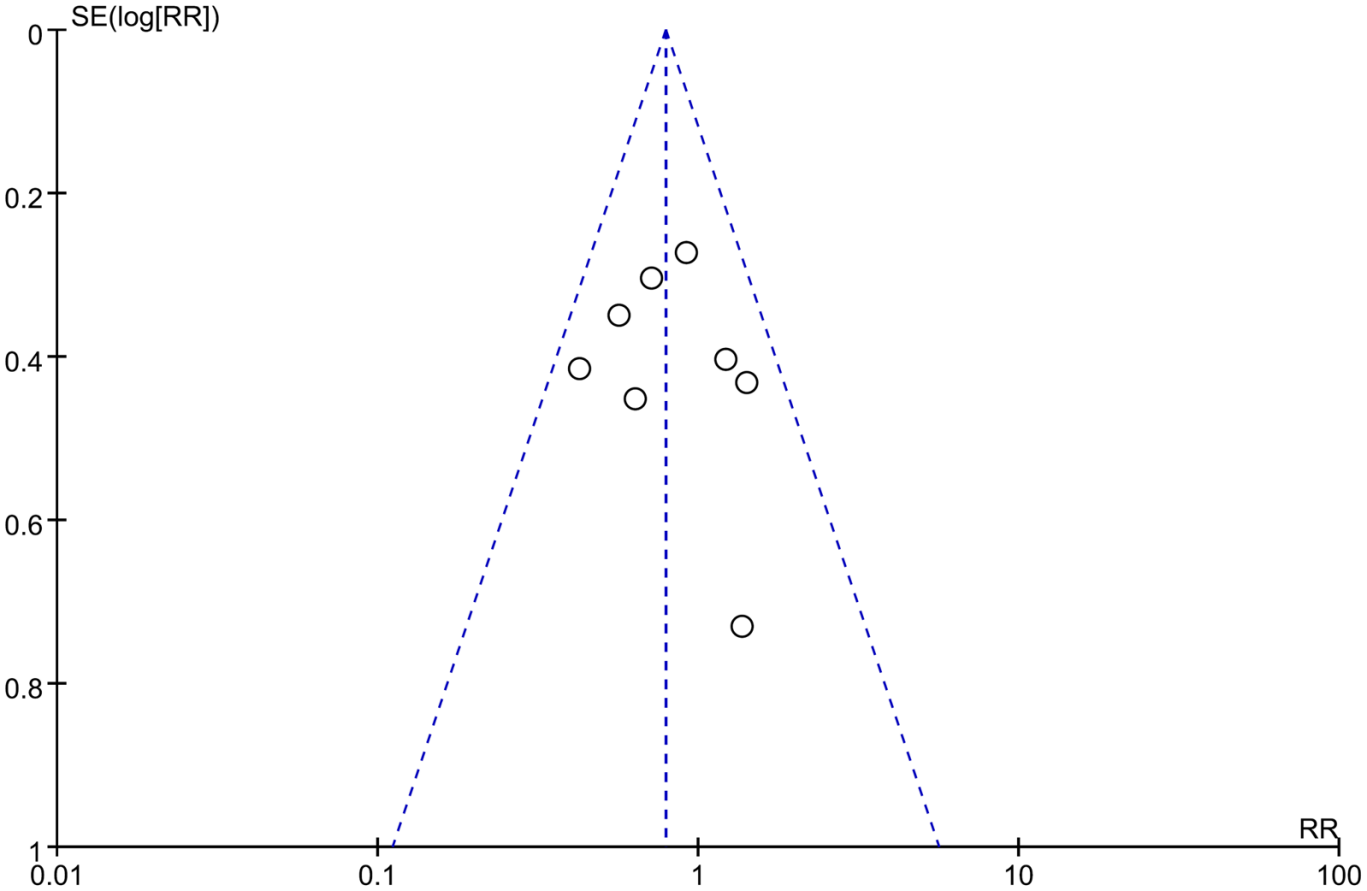
Funnel plot indicating no evidence of heterogeneity between trials for distant metastasis rate. RR values included in the meta-analysis are indicated on the abscissa axis, and the SE (Log OR) values are indicated on the ordinate axis. The impact of publication bias was assessed by observing the symmetry.

In this meta-analysis, the RR value for distant metastasis during follow-up was 0.79 (95% CI: 0.61–1.02, *P* = 0.070) for the aforementioned eight RCTs [8, 14, 23, 24, 26–29] (Fig 4B), with no significant difference (*P* > 0.05) between the CCRT and RT alone groups. The publication bias was small, with the eight spots being substantially symmetric (*t* = 0.43, *P* = 0.685) in Fig 5.

### Sensitivity analysis

Six of the RCT studies included only SCC [23–24, 26–29] and when these were also compared for CCRT and RT, after removing the three studies [8, 14, 25] that included both SCC and AC, the statistical results had no significant difference. These results indicated that the reliability of the analysis was strong.

## Discussion

Progress has been rapid in the CCRT of patients with advanced esophageal cancer, and this may be related to the possible biological mechanisms of chemoradiation and cancer. First, combinatorial radiation and chemotherapy inhibit tumor cell proliferation kinetics with effective radiation control of local disease leading to amplified damage of tumor cells in concurrent therapy [33, 34]. Secondly, chemotherapy can improve microcirculation and reduce the anoxia in cells, thus sensitizing the cells to radiation [35, 36]. Thirdly, chemotherapy inhibits sublethal and potentially lethal repair of tumor cells after radiotherapy. Therefore, the inclusion of chemotherapy during radiation not only enhances the local effects of radiation and thus decreases the likelihood of spread from the primary tumor, it also reduces or eliminates micrometastases [14]. Additionally, when the two forms of therapy are used to intervene at the same time at the beginning of treatment, there is no delay between the treatment of the local lesion and distant metastasis [37]. However, CCRT will continue to face some challenges. More prospective, randomized, stratified phase III studies are needed for further evidence of its efficacy.

Although only two RCTs covered the overall response rate of the primary tumor, it was possible to synthesize the data and the results were shown as a forest plot. The fixed-effect model was used for analysis because there was no heterogeneity (*P* = 0.91, I^2^ = 0%). Due to the small number of samples and the small difference in the overall response rate between CCRT group and RT alone group, the *P* value was 0.05 and the statistical difference was not very significant (Fig 1). Nevertheless, the result still showed that CCRT can increase the overall response rate of the primary tumor. These findings were consistent with the report by Welsh et al. [33], which suggested that chemotherapy can effectively kill tumor cells and rapidly reduce tumor size. Moreover, the chemotherapy drugs in the two identified RCTs with an overall response rate were 5-fluorouracil (5-FU) and capecitabine, which were shown to be more effective at killing local tumor and preventing metastasis

The patient survival rate was increased by CCRT, according to our analysis, which is consistent with other previous reports [8, 13, 14, 25, 29], and thus the synergistic effects of CCRT were confirmed. In addition, the higher doses of radiation (≥50 G) used in six of nine studies might be helpful to survival rates, according to the study by Mirinezhad et al.[25], which indicated that higher doses of radiation were associated with a higher survival rate. However, the results for the 1-year survival rate contrasted with those of Zhao and colleagues who found that the 1-year survival rates of the CCRT and RT alone groups were 67% and 77%, respectively [23]. Different sources of subjects may be the reason for their contrasting findings. Sixty-one percent of patients in our meta-analysis came from Europe, the USA, and other developed countries, whereas patients in studies showing a worse outcome for CCRT were mostly from developing nations. Their nutritional status was worse, which resulted in low patient tolerance for strong chemotherapy regimens and the survival rate was correspondingly lower [38]. It is worth noting that there was a minor portion of patients with adenocarcinoma (2.6%) in three studies, but no further detailed subgroup analysis was performed [8, 14, 25], as only Cooper et al. [8] indicated that combined therapy increases the survival of patients who have either squamous cell or adenocarcinoma of the esophagus, compared with RT alone.

Acute toxic effects of grade 2 or above were more severe in the CCRT group likely due to the types of chemotherapeutic drugs used. A combination of 5-FU and cisplatin is generally the first-line chemotherapy for esophageal carcinoma. However, 5- FU has prominent side effects such as mucosal inflammation and ulceration, leading to severe vomiting or esophageal injury, as previously reported [39]. The toxic effects of chemotherapy and radiotherapy are key factors affecting the planned course of therapy. Overall, 163 patients from the nine studies refused to complete the CCRT due to acute toxicities, leading to a dropout rate of 14.4%. Furthermore, 17 patients (10.4%) died of malnutrition, poor immunity, and hepatic and renal failure, indicating that the acute toxicities from CCRT could increase the risk of death in patients with esophageal cancer. In the later stages of treatment, pulmonary fibrosis, esophageal stenosis, hemorrhage, perforation, and other complications occurred, but there was no significant difference between the two groups (*P* > 0.05). Therefore, clinicians must pay careful attention to the type of CCRT used for treating advanced esophageal cancer. For example, according to some reports [40, 41], there is greater efficacy when paclitaxel is combined with cisplatin in chemoradiotherapy. Additionally, the regimens for radiotherapy are also a concern. Conventional radiation was used in almost all of the studies, and definitive radiotherapy was only found in one study. With the progress in radiotherapy, three-dimensional conformal and definitive radiotherapy can achieve ideal dose distribution and coverage over the target volume, while protecting the normal tissue around the esophageal carcinoma [42, 43]. Consequently, the efficacy is improved and there is some reduction in radiation injury.

Tumor persistence and recurrence were factors in the death of patients with advanced esophageal cancer, and distant metastasis was another factor. Our meta-analysis indicates that CCRT provided local control of the tumor and prevented its recurrence, but the rate of distant metastasis was not significantly different between the two groups (*P* = 0.07), suggesting that death from esophageal cancer was primarily due to distant metastasis. Clinicians should provide appropriate nutritional support to enable patients to undergo two to four cycles of chemotherapy, and thus reduce the incidence of distant metastasis and increase patient survival and quality of life [44, 45].

While potential limitations of this meta-analysis exist, publication bias cannot be avoided due to lack of primary studies. Additionally, patients in the nine studies differed in age, origin, histology type and phase of the tumor, although the majority of patients were above 60 years old. Subgroup analyses were not performed. Rather, the studies were pooled to increase the statistical power of our analyses, and in conclusion, our meta-analysis indicates that CCRT is an effective approach for treating esophageal cancer, based on the improved survival rates and local control rates.

## Supporting Information

S1 File A list of the full-text excluded articles in the supporting information. (DOC)

S1 Checklist PRISMA checklist. (DOC)
