# Supplementary material for: A Meta-Analysis of Concurrent Chemoradiotherapy for Advanced Esophageal Cancer
Source: PLoS One. 2015 Jun 5;10(6):e0128616. doi: 10.1371/journal.pone.0128616 (PMC4457836; doi:10.1371/journal.pone.0128616)
Supplement: S1 File — (DOC) [file pone.0128616.s001.doc]

**A list of the full-text excluded articles in the supporting information**

**A. Patients were in the early stages of cancer：**

(1) Smith TJ, Ryan LM, Douglass HO, Jr., Haller DG, Dayal Y, et al. (1998) **Combined chemoradiotherapy vs. radiotherapy alone for early stage squamous cell carcinoma of the esophagus: a study of the Eastern Cooperative Oncology Group**. Int J Radiat Oncol Biol Phys 42: 269-276.

(2) Wobbes T, Baron B, Paillot B, Jacob JH, Haegele P, et al. (2001) **Prospective randomised study**

**of split-course radiotherapy versus cisplatin plus split-course radiotherapy in**

**inoperable squamous cell carcinoma of the oesophagus.** Eur J Cancer 37: 470-477.

**B. Patients had undergone esophagectomy:**

(1) Kitamura K, Kuwano H, Watanabe M, Nozoe T, Yasuda M, et al. (1995) **Prospective randomized study of hyperthermia**

**combined with chemoradiotherapy for esophageal carcinoma**. J Surg Oncol 60: 55-58.

**(2)** Ma DY, Tan BX, Liu M, Li XF, Zhou YQ, et al. (2014) **Concurrent three-dimensional conformal radiotherapy and chemotherapy for postoperative recurrence of mediastinal lymph node metastases in patients with esophageal squamous cell carcinoma: a phase 2 single-institution study.** Radiat Oncol 9: 28.

**C.** **Outcomes did not include the 1-year and 3-year survival rate, or the rates of recurrence or distant metastasis:**

1. Ji FZ, Zhu WG, Yu CH, Tao GZ, Pan P, et al. (2013) [**A randomized controlled trial of intensity-modulated radiation therapy plus docetaxel and cisplatin versus simple intensity-modulated radiation therapy in II-III stage esophageal carcinoma**]. Zhonghua Wei Chang Wai Ke Za Zhi 16: 842-845.
2. Liu M, Shi X, Guo X, Yao W, Liu Y, et al. (2012) **Long-term outcome of irradiation with or without chemotherapy for esophageal squamous cell carcinoma: a final report on a prospective tria**l. Radiat Oncol 7: 142.
3. Nishimura Y, Hiraoka M, Koike R, Nakamatsu K, Itasaka S, et al. (2012) **Long-term follow-up of a randomized Phase II study of cisplatin/5-FU concurrent chemoradiotherapy for esophageal cancer (KROSG0101/JROSG021).** Jpn J Clin Oncol 42: 807-812.
4. Shan GY, Zhang S, Li GW, Chen YS, Liu XA, et al. (2011) **Clinical evaluation of oral Fructus bruceae oil combined with radiotherapy for the treatment of esophageal cancer**. Chin J Integr Med 17: 933-936..
5. Fallai C, Bolner A, Signor M, Gava A, Franchin G, et al. (2006) **Long-term results of conventional radiotherapy versus accelerated hyperfractionated radiotherapy versus concomitant radiotherapy and chemotherapy in locoregionally advanced carcinoma of the oropharynx**. Tumori 92: 41-54.
6. Slabber CF, Nel JS, Schoeman L, Burger W, Falkson G, et al. (1998) **A randomized study of radiotherapy alone versus radiotherapy plus 5-fluorouracil and platinum in patients with inoperable, locally advanced squamous cancer of the esophagus**. Am J Clin Oncol 21: 462-465.
7. Ogoshi K, Satou H, Isono K, Mitomi T, Endoh M, et al. (1995) **Immunotherapy for esophageal cancer. A randomized trial in combination with radiotherapy and radiochemotherapy. Cooperative Study Group for Esophageal Cancer in Japan.** Am J Clin Oncol 18: 216-222.
8. Hurt CN, Nixon LS, Griffiths GO, Al-Mokhtar R, Gollins S, et al. (2011) S**COPE1: a randomised phase II/III multicentre clinical trial of definitive chemoradiation, with or without cetuximab, in carcinoma of the oesophagus**. BMC Cancer 11: 466..
9. van Heijl M, Phoa SS, van Berge Henegouwen MI, Omloo JM, Mearadji BM, et al. (2011) **Accuracy and reproducibility of 3D-CT measurements for early response assessment of chemoradiotherapy in patients with oesophageal cancer**. Eur J Surg Oncol 37: 1064-1071.
10. Zhou JC (1991) **Randomized trial of combined chemotherapy including high dose cisplatin and radiotherapy for esophageal cancer**. Zhonghua Zhong Liu Za Zhi 13: 291-294.

**D. Studies had fewer than 50 samples:**

1. Lin Q, Gao XS, Qiao XY, Chen K, Wang YD, et al. (2008) [Phase II clinical trial of concurrent chemoradiotherapy (cisplatin plus 5-fluorouracil) for esophageal cancer]. Ai Zheng 27: 1077-1081.
2. Kaneta T, Takai Y, Nemoto K, Kakuto Y, Ogawa Y, et al. (1997) **Effects of combination chemoradiotherapy with daily low-dose CDDP for esophageal cancer--results of a randomized trial**. Gan To Kagaku Ryoho 24: 2099-2104.

**E. Duplicate study:**

(1) al-Sarraf M, Martz K, Herskovic A, Leichman L, Brindle JS, et al. (1997) **Progress report of**

**combined chemoradiotherapy versus radiotherapy alone in patients with esophageal cancer: an intergroup study**. J Clin Oncol 15: 277-284.
